# MeSH and text-word search strategies: precision, recall, and their implications for library instruction

**DOI:** 10.5195/jmla.2022.1283

**Published:** 2022-01-01

**Authors:** Michelle M. DeMars, Carol Perruso

**Affiliations:** 1 michelle.demars@csulb.edu, Health Sciences Librarian, California State University, Long Beach, Long Beach, CA; 2 carol.perruso@csulb.edu, Associate Librarian, California State University, Long Beach, Long Beach, CA

**Keywords:** precision, recall, MeSH, MeSH instruction, relevant recall method

## Abstract

**Objective:**

This study compared the recall and precision of MeSH-term versus text-word searching to better understand psychosocial MeSH terms and to provide guidance on whether to include both strategies in an information literacy session or how much time should be spent on teaching each search strategy.

**Methods:**

Using the relevant recall method, a total of 3,162 resources were considered and evaluated to form a gold standard set of 1,521 relevant resources. We compared resources discussing psychosocial aspects of children and adolescents living with type 1 diabetes using two search strategies: text-word strategy versus MeSH-term strategy. The frequency of MeSH terms, the MeSH hierarchy, and elements of each search strategy were also examined.

**Results:**

Using the 1,521 relevant articles, we found that the text-word search strategy had 54% recall, while the MeSH-term strategy had 75% recall. Also, the precision of the text-word strategy was 34.4%, while the precision of the MeSH-term strategy was 47.7%. Therefore, the MeSH-term search strategy yielded both greater recall and greater precision. The MeSH strategy was also more complicated in design and usage than the text-word strategy.

**Conclusions:**

This study demonstrates the effectiveness of text-word and MeSH search strategies on precision and recall. The combination of text-word and MeSH strategies is recommended to achieve the most comprehensive results. These results support the idea that MeSH or a similar controlled vocabulary should be taught to experienced and knowledgeable students and practitioners who require a myriad of resources for their literature searches.

## INTRODUCTION

For clinicians, health librarians, and students alike, conducting effective and efficient literature searches is an important part of evidence-based medicine (EBM). Sackett, who is considered one of the pioneers of evidence-based medicine, defines EBM as “the conscientious, explicit, and judicious use of current best evidence in making decisions about the care of individual patients” [1]. It is this desire to effectively and safely treat patients, using well-documented methods, that has health practitioners and health students in a continuous search for the best evidence and most relevant literature. EBM recommends consulting articles on randomized controlled trials or systematic reviews. This need for specific types of literature can influence a search strategy and adds to the skills needed to accomplish a successful search.

Effective searching to find relevant literature is a complex skill that is gradually learned and goes beyond many of the databases undergraduates are introduced to in their general education classes and well beyond Google or Google Scholar [2]. Nor is it something that can be mastered in a one-shot information literacy session. It requires more than a basic knowledge of common medical databases, each with different interfaces and controlled vocabularies. Information literacy competence for nursing students, as defined by the Association of College & Research Libraries, includes five standards with more than 130 outcomes and skills to be mastered. Addressing these standards, while dealing with the wide variety of skills students bring to the one or two sessions incorporated in their undergraduate studies, requires significant prioritizing. Librarians' hope is that students learn how, as practitioners, they can conduct effective searches as they pursue the goal of finding “as much information as is available on a specific topic and … as few articles as possible that are unrelated to the search topic” without becoming overwhelmed [3].

A health sciences librarian who teaches students to conduct literature searches frequently weighs whether to include both strategies or how much time should be spent teaching text-word searching versus subject heading searching. While working with two researchers, the question of the most effective search strategy surfaced, leading us to the present study comparing the precision and recall of Medical Subject Headings (MeSH) and text-word searching in PubMed and three other databases that use a similar controlled vocabulary, though the tree below each term may vary.

Even though many researchers have tackled this instructional challenge, with “evidence to suggest a positive relationship between library instruction and information literacy skill development” and multiple recommendations for “sustained training and support across year levels,” we could find no studies that specifically address teaching students the intricacies of selecting the best MeSH terms, a skill that taxes even experienced academic librarians and has generated a plethora of skills tutorials [4–13].

Yet nursing and other health care faculty naturally want students to learn the language and landscape of the medical literature, including MeSH. Such a request from a nursing professor, combined with helping two researchers with a scoping review, led to the current study. One of the aims of this study was to test whether MeSH or text-word searching (or a combination) was most effective in researching psychosocial phenomena in adolescents. The other goal, which involved converting text-words into comparable MeSH terms, was to observe and document our own process with an eye toward balancing the teaching of text words and MeSH or a similar controlled vocabulary in undergraduate and graduate information literacy instruction. For the first goal, we used recall and precision, two long-standing bibliometric measurements of the effectiveness of search strategies that continue to be used, even as advances in automated text retrieval have produced other evaluation methods [14–20].

Recall is defined “as the number of relevant citations retrieved by a search divided by the number of relevant citations” [21]. For example, if a search retrieves 100 documents, 75 of which are relevant to the research question, but misses another 25 relevant documents, then the recall of the search (75 retrieved relevant documents/125 total relevant documents) is 60%. Researchers strive to retrieve the most relevant articles possible without missing any important resources [22]. The benefit to higher recall is the breadth of coverage, while the challenge lies in the time required to examine each result. (Sensitivity can be an alternative term for recall when evaluating information retrieval.) The other factor, precision, is defined as “the number of relevant citations retrieved divided by the total number of citations retrieved” [21]. Using the same example, if the search retrieves 100 documents, 75 of which are relevant to the research question, the search's precision (75 relevant documents/100 retrieved documents) is 75%. This factor represents fewer, more focused results, with the goal to gather few articles that are unrelated to the topic [3]. The benefit to high precision is the exactitude of the results and the time saved in evaluating them, with the challenge being potentially missing relevant articles.

Many elements can affect the precision and recall of a literature search, and a researcher's search strategy is an important part of the equation. Other elements include the quality, quantity, and relevance of the articles in chosen databases. The two primary strategies commonly used by researchers are text-words or keywords and subject headings from controlled vocabulary. While we consider text-words and keywords to be interchangeable, the medical research literature uses text-words almost exclusively. However, this is different from the PubMed field Text Word [TW], which searches “all words and numbers in the title, abstract, other abstract, MeSH terms, MeSH Subheadings, Publication Types, Substance Names, Personal Name as Subject, Corporate Author, Secondary Source, Comment/Correction Notes, and Other Terms” [23]. With text-word or keyword searching, researchers generate their own search terms based on their topic and their knowledge of the vocabulary used by the discipline. Text-words are often used as a “substitute for a subject search when [the searcher does] not know the standard subject heading” [24]. They may be used to search the full text or portions of the record, such as the title and abstract of an article. (In addition to MEDLINE citations, PubMed includes in-process and “ahead of print” citations yet to be indexed with MeSH, out-of-scope general science and general chemistry journals, some author manuscripts, and NCBI books [25].) Subject searching uses controlled vocabulary “from a predetermined list of possible terms [assigned to] reflect the content of the item” [23]. MeSH terms are an example of a controlled vocabulary assigned by the National Library of Medicine to the article citations in MEDLINE and most PubMed content, and a similar controlled vocabulary is also used in some form in several other databases, such as CINAHL. Research indicates that a combination of strategies is the best approach [3, 22, 26, 27, 28].

Specifically, this study compares these two methods while researching psychosocial factors in children and adolescents with type 1 diabetes. This particular topic was chosen because of the collaboration with two researchers, Bell (an assistant professor at California State University, Long Beach) and Hazel (a clinical social worker in the Division of Endocrinology at Boston Children's Hospital), and because researching psychosocial factors in PubMed can present greater challenges than searching biomedical terms, for which PubMed has specific search tools. Because it is a broad topic, yet typical of one that health science students would undertake, we believed that it lent itself to an investigation of the pluses and minuses of MeSH (or similar controlled vocabulary) and text-word strategies.

There is no shortage of literature discussing various aspects of MeSH, including search strategy differences by type of user and the use of MeSH for literature searches. The comparison of text-word versus controlled vocabulary, such as MeSH, has also been discussed in the literature for many years, as have the challenges MeSH and similar controlled vocabulary present, especially to inexperienced searchers. While the prevalent thinking is that a combination approach is best [3, 22, 26, 27, 28], going back to the mid-1990s, Lowe and Barnett recognized that MeSH was not frequently utilized by health care professionals because of its complicated nature and lack of availability to those outside the library field [21].

Haynes and colleagues added to this early discussion with their study on developing search strategies with a focus on MEDLINE [26]. Their study outlined the challenges of balancing precision and recall while developing a search strategy, and their results showed that precision and recall were enhanced by combining MeSH and text-word searching.

The conversation continued nearly a decade later with studies on otolaryngology and sleep. Both Jenuwine and Floyd and Chang, Heskett, and Davidson found that text-word searching produced a higher number of results but did not exclude irrelevant articles very well [22,27]. Both studies concluded that thorough researchers should use a combination of strategies, especially when a comprehensive and broad search is required, such as for a systematic review.

Comparisons of the usage of MeSH and text-word search strategies open the door to a deeper conversation about how MeSH is constructed and if an understanding of this structure will lead to searches that are both precise and comprehensive. Gault, Shultz, and Davies sought to compare the mapping of MeSH across a variety of interfaces including the MeSH Browser and OVID [29]. Their study revealed inconsistencies in the results of the MeSH term associated with the search term, depending on the interface used. They also found that the interface selected could affect the search results even if each was mapping to MeSH. Richter and Austin contributed to the discussion with a report reviewing how MeSH and text-words are used to search for literature in PubMed and how text-words are mapped to a MeSH term [28]. By using example searches from the field of physical therapy, the authors searched PubMed for both search terms and acronyms to determine if the item entered mapped to MeSH terms. Slightly less than half of the terms mapped appropriately, and the remaining terms mapped inappropriately or not at all. This issue emphasizes the benefits of text-word searching as an alternative or additive to MeSH searching.

Given the evidence that both search strategies have their merits, librarians are faced with the question of how much of their limited instruction time should be dedicated to teaching each search strategy. The struggle to use valuable instruction time on MeSH (or similar controlled vocabulary) led to discussions between a health science librarian and nursing faculty about the perceptions of how nursing students were grasping text-word and MeSH search strategies as demonstrated in their coursework and assignments. There were commonalities among faculty observations, especially as they compared search strategies among undergraduates and early master's of science in nursing students to those of more experienced graduate students and doctor of nursing practice (DNP) students. Nursing faculty find that undergraduates will seek out the “path of least resistance” when it comes to their literature searches, often depending on text-word searching as that is what they are most familiar with [N. Cheffer, email to M. DeMars, July 6, 2021]. One faculty member noted, “Most [undergraduate] students use CINAHL which requires an initial first step to choose MeSH searches … and therefore students tend to settle too quickly for the keyword searches.” Comparatively, nursing faculty noticed that more experienced students, such as those in the DNP program, many of whom are already nurse practitioners, are more likely to grasp the concepts of MeSH and use it more frequently in both PubMed and CINAHL. Also noted by faculty was this population's awareness of the benefits of a more comprehensive search strategy in relation to preparing a manuscript for publication: “[DNP students] aspire to publish and know that identifying the MeSH terms they used is evidence of more professional literature searches” [AJ Jadalla, email to M. DeMars, July 7, 2021].

These nursing faculty observations highlight important distinctions between the two student populations. Inexperienced and undergraduate students are less likely to embrace MeSH, as they are still working to grasp clinical concepts and are less likely to need to justify their search process. Experienced and doctoral students are more likely to welcome MeSH search strategies and may already be using them for their work or practice. Additionally, their drive to publish a manuscript as part of their rigorous academic coursework may have this population more willing to learn the intricacies of MeSH for their assignments. The complicated hierarchy and tree structure of MeSH is often overshadowed by the popularity of text-word or keyword searching with its ease of use and the speed of finding results. MeSH terms are complicated in comparison, especially to inexperienced researchers, and therefore may be left out of an instructional session by health science librarians. Health practitioners also experience difficulties with MeSH, with search errors commonly related to the MeSH mapping structure [30]. These various complexities call into question if the additional results outweigh the time expended.

## METHODS

For practitioners, finding all relevant research is important, but rarely are they able to take the time-consuming steps necessary to create a “gold standard” list of sources “of known relevance to the concept … which when considered cumulatively, should ideally represent the full scope of that concept” [31]. Research shows that it is not unusual for this process to take in excess of 100 hours to gather and requires an expert searcher even more than a domain expert [32,33]. Such a complete list of relevant research is also a necessary first step to measuring recall. Completeness is never perfect, as it is limited not only by time but also by the sources searched, the quality of the search strategy, as well as any subjective bias in the search strategy or from evaluators. Researchers have used combinations of processes, including hand searching relevant journals, mining systematic reviews, searching multiple databases, searching grey literature, checking cited references, and expert or other qualitative evaluation [3, 19, 31, 34, 35]. When a hand search is not practical, some researchers use a method called relative recall [31,36,37]. Relative recall combines “multiple exhaustive and high-quality searches across a broad range of sources, as well as a rigorous screening process based on clear eligibility criteria … [to] minimise the potential for bias” [31]. In contrast to recall, precision is a relatively straightforward measure, with accuracy dependent on the comprehensiveness of the search strategy and the time needed to review results to determine the proportion that are relevant.

For this study comparing the recall and precision of MeSH terms (or similar controlled vocabulary) versus text-word searching, the relevant-recall method was used to form the gold standard set of resources. Building upon the resources Bell and Hazel [38] found using a text-word-only Boolean strategy in nine databases (Academic Search Premier, CINAHL, Dissertations & Theses, Embase, Global Health, LWW Nursing, PsycInfo, PubMed, and Web of Science) (Appendix B), we created parallel MeSH-only Boolean searches of PubMed, CINAHL, Embase, and PsycInfo (Appendix A), which are four databases that include the option of searching with MeSH terms or a similar controlled vocabulary. (See Appendix C for a comparison.) The MeSH search builder was used in the version of PubMed launched in spring 2020 to generate the search string, thereby bypassing nonindexed records. Both search strategies aimed to identify relevant research defined by Bell and Hazel as studies that included instruments measuring “individual and family factors … related to self-perception, interpersonal factors, and individual responses” of youth living with type 1 diabetes [38]. Given the nature of the MeSH tree structure and the hierarchy of terms, the MeSH terms used for the searches also included any terms that were categorized below them in the MeSH tree [39]. The MeSH terms in this default setting allow for the inclusion of the term, plus some that are related, resulting in broad results related to that term, a method known as MeSH explosion. In addition to their text-word searches, Bell and Hazel mined 14 systematic reviews and references in relevant resources for additional sources, which were added to the combined set [38]. Otherwise, we did not conduct hand searches, although Bell and Hazel conducted a few. The combination of these strategies yielded 3,162 sources, with 1,375 coming from the text-word search plus reference mining and 1,787 coming from the MeSH-term (or similar controlled vocabulary) search.

To refine this collection of resources and to document our process, we created a master spreadsheet showing which database and search method yielded each source. Prior to eliminating any articles, we noted MeSH terms for each, where available. We conducted multiple levels of evaluation to achieve the final list, first of titles, then of abstracts, and finally evaluation of the full text of the remaining sources. We then eliminated overlap between text-word and MeSH-term search results, resulting in 2,378 unique, English-language sources published from January 1, 2010, to July 7, 2020, from the two search strategies. We added ten sources found using cited references or mining systematic reviews for a total of 2,388 sources.

To further refine this set of resources to keep only sources of “known relevance to the concept” of psychosocial factors facing children and adolescents with type 1 diabetes, we employed three methods:

- Reviewing MeSH terms for the articles, building upon the expertise of the National Library of Medicine, whose indexers assign the terms;
- Reviewing titles and abstracts; and
- Reviewing the list of sources that Bell and Hazel ultimately selected for their narrower study, along with the reasons that items were excluded [38].

For this process, we divided the list of 2,388 articles in half, with each author evaluating items independently, conferring with each other or with Bell and Hazel as needed. This more independent approach aimed to emulate the text-word approach and was possible because of Bell and Hazel's expertise with the topic and because their search-result evaluation happened first, giving us a greater level of confidence [38]. Adding to the rigor of the review was one of the authors' expertise as a health sciences librarian and experience as an assistant clinical research coordinator at a major medical center.

**Figure 1:**
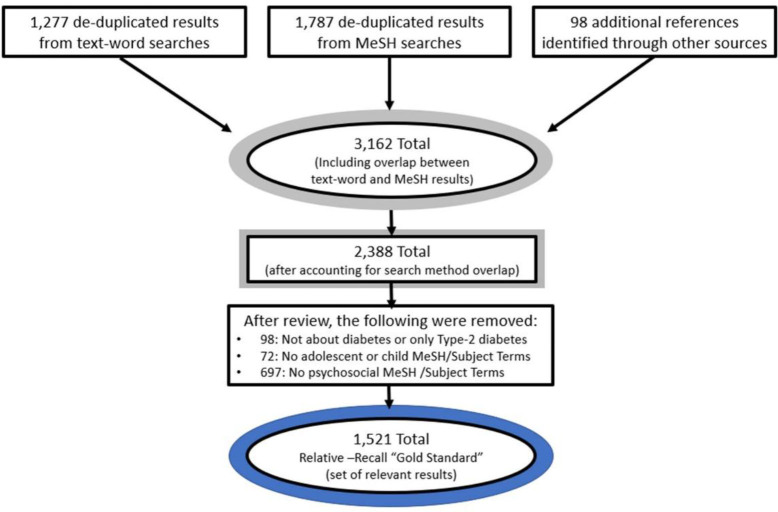
Flowchart of evaluation process

We started by eliminating ninety-eight articles without the MeSH terms or subject headings of “Diabetes Mellitus” or “Diabetes Mellitus, Type 1” or that had only “Diabetes Mellitus, Type 2.” In addition to relying on MeSH terms, we evaluated article titles and/or abstracts. Only four of those eliminated came from the MeSH-only search strategy. Next, we eliminated seventy-two articles that did not have an “Adolescent” or “Child” assigned MeSH term. In addition, we reviewed article titles, abstracts, or the full text to confirm that the studies were about only adults. Studies about adults were kept if they also had “Adolescent” or “Child” MeSH terms or if the studies included participants in both age groups. Sources were only discarded if both authors agreed. We then evaluated the remaining 2,218 articles to determine whether they were relevant to psychosocial factors affecting children and adolescents with type 1 diabetes. Before doing this, we reviewed MeSH terms assigned to these articles to determine whether we needed to expand the number of psychosocial MeSH terms beyond the 23 terms used in our Boolean search. We added any MeSH terms with a subheading of “Psychology” (i.e., “/Psychology”) plus 13 terms that were assigned to relevant articles and that could have improved the original Boolean search strategy (Table 1). For this step, 697 articles without at least one of the psychosocial MeSH terms on the list were eliminated. This left 1,521 articles and dissertations in the gold standard list. It also provided us with the relevant sources organized by database to measure the precision of two search strategies (Figure 1).

**Table 1:** MeSH terms used to evaluate source relevance

MeSH terms in initial search		Added MeSH terms while determining gold standard
Burnout, Psychological	Psychology	Any term with “/Psychology”
Family Conflict	Psychology, Adolescent	Adaptation, Psychological
Fear	Psychosocial Support Systems	Adolescent Behavior
Health Communication	Self Care	Anxiety
Hope	Self Concept	Depression
Optimism	Self Efficacy	Emotional Adjustment
Patient Acceptance of Health Care	Social Isolation	Emotions
Patient-Centered Care	Social Stigma	Health Behavior
Perception	Social Support	Health Knowledge, Attitudes, Practice
Professional-Patient Relations	Teach-Back Communication	Impulsive Behavior
Psychological Distress	Uncertainty	Motivation
		Parent-Child Relations
		Stress, Psychological
		Quality of Life

The amount of time this process took would weigh heavily on practitioners and students. Similar to the amount of time for such searching found by others, the two authors (as well as Bell and Hazel) *each* spent in excess of 100 hours before reaching their final lists. Even with a well-constructed search combining the two methods, evaluating results would have consumed well more than 100 hours [33].

## RESULTS

**Figure 2:**
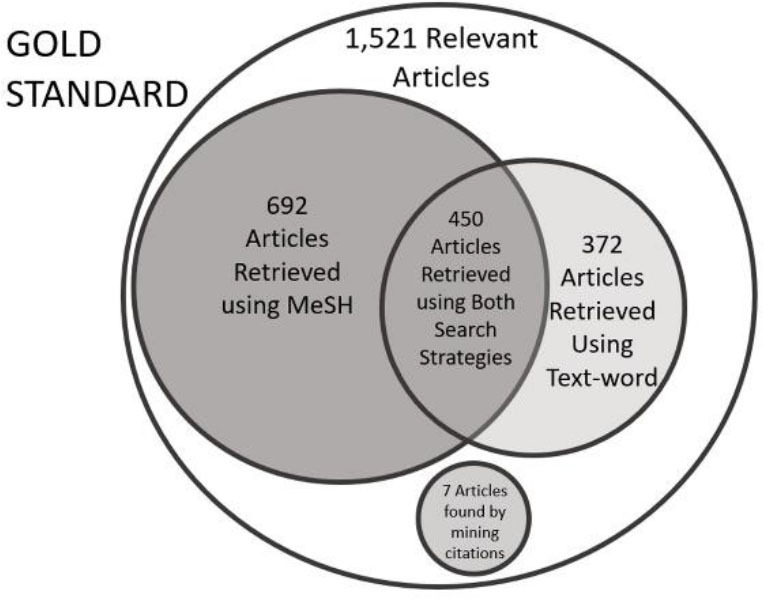
Relationship of sources found by each method

Of the 1,521 relevant articles and dissertations, 372 were found only using the text-word search strategy, 692 were found only using the MeSH-term strategy, and 450 were found with both strategies. An additional seven results were found only by mining citations (Figure 2). Using the 1,521 relevant articles and dissertations as the denominator for the recall formula used by Ting [40], we found that the text-word search strategy had 54% recall (822 retrieved/1,521 relevant sources), while the MeSH-term strategy had 75% recall (1,139 retrieved/1,521 relevant sources).

To measure the precision of each method, we used Ting's method and divided the number of sources retrieved by each method by the total number of sources retrieved by the two search methods combined [40]. For this step, we removed eight sources found manually. Thus, the precision of the text-word strategy was 34.4% (822 relevant sources retrieved/2,388 total unique sources), while the precision of the MeSH-term strategy was 47.7% (1,139 relevant sources retrieved/2,388 unique sources). Therefore, the MeSH-term search strategy yielded both greater recall and greater precision.

The disparity widened when we compared the two search strategies for the 1,367 articles that appeared in the only freely available database, PubMed. The text-word strategy yielded 49.8% of the articles, while the MeSH-term strategy produced 81.4%. The greater recall and greater precision may have been influenced by the automatic explosion of the MeSH terms.

However, despite higher recall and precision for the MeSH-term strategy, there were 236 sources in the gold standard set (15.5%) that had no MeSH terms assigned or were not indexed in PubMed and were only found using the MeSH-term strategy in another database. All but twenty-two of those could be discovered only using the text-word strategy or through reference mining.

Furthermore, we calculated the recall of each search strategy for the four databases. The MeSH-term strategy yielded:

- 60.2% recall in PubMed (916 of 1,521 relevant sources)
- 24% recall in Embase (365 sources)
- 17.8% recall in CINAHL (269 sources)
- 11.6% recall in PsycInfo (177 sources)

The text-word search strategy yielded:

- 34.2% recall in Embase (520 of 1,521 relevant sources)
- 32.8% recall in PubMed (499 sources)
- 28.5% recall in CINAHL (433 sources)
- 19.9% recall in PsycInfo (303 sources)
- 8.9% recall in Academic Search Premier (136 sources)

The MeSH term strategy was most effective in PubMed, while Embase and PubMed were nearly tied in the text-word strategy. For PsycInfo and CINAHL, the text-word strategy was more effective than the MeSH-term strategy. Also of note:

- Eighty-five sources appeared in PubMed that neither search strategy located but were found in other databases.
- The MeSH-term Boolean strategy in PubMed missed 135 sources with appropriate MeSH terms. Of these, all but nineteen were found using the text-word strategy.
- The MeSH-term Boolean strategy yielded 287 sources in PubMed that had none of the MeSH terms used in the strategy. Of these, the text-word strategy missed 229.

**Figure 3:**
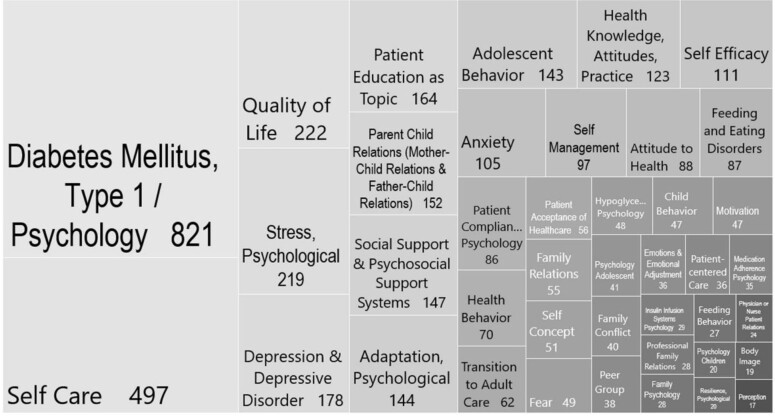
Frequency of assigned MeSH terms

Finally, we examined the most frequent MeSH terms and concepts to aid in future search strategies. For this, we grouped a few similar terms. “Diabetes Mellitus, Type 1/Psychology” was overwhelmingly the most frequent, assigned to 821 of the 1,281 articles with MeSH terms in PubMed. (“Diabetes Mellitus, Type 1” was included in the MeSH-term strategy, but this count is only for those with the subheading “Psychology.”) Among the top 40 concepts, several frequently assigned terms of note were not part of the MeSH-term search strategy (Figure 3).

## DISCUSSION

The results of this study reaffirm that the MeSH-term search strategy yields both greater recall and greater precision. Therefore, even though the difficulty level of using MeSH (or similar controlled vocabulary) to search for literature may be higher, there is a substantial benefit to using MeSH as part of an effective search strategy. The benefit is especially pronounced when a researcher is conducting an exhaustive literature search, as would be the case for a systematic review. The search results that MeSH provides can support the needs of health practitioners looking to find high-quality evidence as part of their EBM practices and students seeking publication. This study also supports the prevalent thinking that using a combination of MeSH and text-word strategies is beneficial. Although the MeSH strategy provided high precision and recall, it missed a substantial number of resources in PubMed (15.5% in this study) as not all sources were indexed using MeSH terms, either because they were not selected for indexing or because of the time needed for indexing [41]. Additionally, we found that for at least two important databases (CINAHL and PsycInfo), text-word searching was more effective. Therefore, the best practice would include a text-word search to catch any resources that would otherwise be missed because of their lack of indexing and to add a search of non-MeSH databases to catch any additional resources. Many combinations can accomplish gathering both indexed and nonindexed literature. One strategy is the combination of MeSH and text-words in a singular search [35], and another is performing two separate searches—one MeSH and one text-word—and then combining the results. If researchers are performing a quick search or only require a singular article for an assignment, then integrating MeSH may not be as important. However, if they are doing extensive and comprehensive searches such as systematic reviews, adding MeSH will provide greater coverage.

Even with the demonstrated benefits of MeSH (or similar controlled vocabulary) and the recommendations of a combined MeSH and text-word approach, a medical or health sciences librarian should consider the needs of their student population when including basic MeSH strategies in their curriculum. Librarians should consider the experience level of the students they will be teaching and the publishing goals of the students. Those who are considering publication may be drawn to MeSH as a search strategy to strengthen their manuscript. As highlighted by the observations of nursing faculty, for beginners and undergraduate students, learning the complexities of MeSH may prove to be too time-consuming and may even hit some resistance from this population, especially considering the likelihood that their assignments would not require more than a few relevant articles. One strategy tested with some success in a Canadian nursing program ramped up information literacy instruction over three years by teaching basic CINAHL searching and popular and scholarly literature differences in year one, advanced CINAHL searching and critical website evaluation in year two, and formulating a research question and searching PubMed using both MeSH and clinical queries in year three [4].

For doctoral and experienced graduate students, however, a librarian may consider MeSH to be an essential component of their research curriculum. By focusing on the basics of MeSH terms and search strategies, a medical or health sciences librarian can provide students and current and future practitioners with a beneficial edge to their research strategies [42]. It is our recommendation that the basics should include not only skills-based information but also an introduction to the structure, mapping, and functionality of MeSH, as the intricacies of MeSH hierarchies can impact their effectiveness as a search strategy. For example, if a text-word term is searched in PubMed, one can explore how the database interprets and maps that term by exploring the history and search details sections. This area of the interface allows researchers to evaluate the MeSH terms associated with their text-word terms without directly interacting with the MeSH search tool, helping students understand the structure of MeSH terms in a way that is familiar. From a teaching perspective, this strategy is quick to demonstrate and easy to integrate into the librarian's curriculum, making it an appropriate introduction to the strategy. By providing experienced searchers with instruction in MeSH, librarians can provide them with a more comprehensive search approach that will support assignments and future manuscript publication opportunities. Instructors should also advise graduate students of the importance of combining text-word and MeSH-term strategies, as research repeatedly recommends.

Overall, this study demonstrates the impact of text-word and MeSH search strategies on the precision and recall of search results and hence their importance to instruction. The combination of text-word and MeSH strategies provides the most comprehensive results, and the use of MeSH provides the most precise results. However, the complexities of MeSH and skills needed to master it may only be needed by experienced and knowledgeable students and practitioners who require a myriad of resources for their research. Additionally, by exploring diabetes, a topic that many health sciences students choose to write about, and one for which we could find no previous study of recall and precision, the hope was to assist future researchers in this important field of adolescent health. The recent welcome addition of two MeSH terms, “Psychosocial Intervention” and “Psychosocial Functioning” should make future research much easier.

Several limitations should be considered regarding this study. It should be noted that our research was biased toward the Bell and Hazel approach as it was their desire to generate a scoping review that aided in propelling this study forward. This may have influenced the fact that only fourteen of the sources found using the MeSH-only strategy were ultimately added to the Bell and Hazel study. This study was also limited because very little hand searching was used. Additionally, the vast majority of results for the gold standard list were limited to articles currently indexed using MeSH within each database (fifty-seven sources not assigned MeSH terms in any of the databases were found using text words only). We recognize that some vendors have adapted the MeSH controlled vocabulary for their products. The results were also skewed to those published in the English language as we are fluent only in English. The success of both the text-word and MeSH-term strategies was also limited by the quality of the search strategies, as evidenced by the fact the MeSH term “Adaptation, Psychological” was assigned to 144 sources but was not included in the search strategy.

Future research regarding MeSH and information literacy instruction should explore how librarians and researchers can effectively use and teach MeSH terms. An additional study surveying the practices and trends of health science and medical librarians teaching MeSH would expand on the relationship between MeSH and information literacy instruction and could shed light on how many librarians are teaching MeSH search strategies and to what level of student. An additional study building off our current research could compare current search results and those from a MeSH-term subject heading combination, “Diabetes Mellitus, Type 1/Psychology.” The concept behind this potential future study was inspired by the surprising number of results that included this pairing. This comparison may provide insight into effective strategies that are easy enough for beginning researchers but effective enough to utilize MeSH to its fullest. Additional future research could be developed to gain a better understanding of why there were results that seemed out of place or results that should have been found but were missed.

## Data Availability

Data associated with this article are available in ScholarWorks at http://hdl.handle.net/20.500.12680/kk91fr54x.

## SUPPLEMENTAL FILES

**Appendix A.** MeSH-term search strategies

**Appendix B.** Text-word search strategies

**Appendix C.** Comparison of MeSH terms used
