# Supplementary material for: MeSH and text-word search strategies: precision, recall, and their implications for library instruction
Source: J Med Libr Assoc. 2022 Jan 1;110(1):23–33. doi: 10.5195/jmla.2022.1283 (PMC8830400; doi:10.5195/jmla.2022.1283)
Supplement: Supplementary file 1 — Appendix A. MeSH-term search strategies [file jmla-110-1-23-s01.docx]

**Appendix A: MeSH-Term Search Strategies**

**PubMed —**

((("Diabetes Mellitus, Type 1"[Mesh]) AND ( "Child"[Mesh] OR "Adolescent"[Mesh] )) AND ( "Patient-Centered Care"[Mesh] OR "Self Concept"[Mesh] OR "Hope"[Mesh] OR "Health Communication"[Mesh] OR "Social Stigma"[Mesh] OR "Family Conflict"[Mesh] OR "Uncertainty"[Mesh] OR "Social Support"[Mesh] OR "Social Isolation"[Mesh] OR "Self Care"[Mesh] OR "Perception"[Mesh] OR "Fear"[Mesh] OR "Psychological Distress"[Mesh] OR "Burnout, Psychological"[Mesh] OR "Optimism"[Mesh] OR "Psychosocial Support Systems"[Mesh] OR "Patient Acceptance of Health Care"[Mesh] OR "Psychology"[Mesh] OR "Teach-Back Communication"[Mesh] OR "Psychology, Adolescent"[Mesh] OR "Self Efficacy"[Mesh] )) NOT "Diabetes, Gestational"[Mesh]

**PSYCInfo*—**

MA diabetes mellitus type 1 AND MA ( (child OR adolescent) ) AND MA ( (Self Efficacy OR Psychology, Adolescent OR Teach-Back Communication OR Health Communication OR Self Concept OR Patient Acceptance of Health Care OR Psychological Distress OR Uncertainty OR Hope OR Optimism OR Social Stigma OR Social Isolation OR Social Support OR Psychosocial Support Systems OR Psychosocial Support Systems OR Self Care OR Fear OR Patient-Centered Care OR Family Conflict OR Perception OR Professional-Patient Relations) ) NOT MA "Diabetes, Gestational"

**CINAHL*—**

(MM "Diabetes Mellitus, Type 1") AND (MH "Child+") OR (MH "Adolescence+") AND (MH "Self-Efficacy") OR (MH "Psychology+") OR (MH "Adolescent Psychology") OR (MH "Self Concept+") OR (MH "Psychological Distress") OR (MH "Uncertainty") OR (MH "Hope") OR (MH "Optimism") OR (MH "Stigma") OR (MH "Social Isolation+") OR (MH "Support, Psychosocial+") OR (MH "Self Care+") OR (MH "Patient Centered Care") OR (MH "Fear+") OR (MH "Family Conflict") OR (MH "Perception+") OR (MH "Professional-Patient Relations+") NOT (MH "Diabetes Mellitus, Gestational")

**Embase*—**

'insulin dependent diabetes mellitus'/exp AND ('child'/exp OR 'adolescent'/exp) AND ('clinical study'/exp OR 'questionnaire'/exp) AND ('self concept'/exp OR 'psychology'/exp OR 'child psychology'/exp OR 'interpersonal communication'/exp OR 'medical information'/exp OR 'patient attitude'/exp OR 'distress syndrome'/exp OR 'uncertainty'/exp OR 'hope'/exp OR 'optimism'/exp OR 'social stigma'/exp OR 'social isolation'/exp OR 'social support'/exp OR 'psychosocial care'/exp OR 'burnout'/exp OR 'self care'/exp OR 'patient care'/exp OR 'family conflict'/exp OR 'perception'/exp OR 'professional-patient relationship'/exp) NOT 'pregnancy diabetes mellitus'/exp

*These databases use a similar controlled vocabulary but the tree structure may vary from MeSH
