# Supplementary material for: MeSH and text-word search strategies: precision, recall, and their implications for library instruction
Source: J Med Libr Assoc. 2022 Jan 1;110(1):23–33. doi: 10.5195/jmla.2022.1283 (PMC8830400; doi:10.5195/jmla.2022.1283)
Supplement: Supplementary file 2 — Appendix B. Text-word search strategies [file jmla-110-1-23-s02.docx]

**Appendix B: Text-Word Search Strategies**

**PubMed —**

((((“type 1 diabet*”[Title/Abstract] OR “insulin dependent diabetes”[Title/Abstract] OR IDDM[Title/Abstract] OR “juvenile diabetes”[Title/Abstract] OR “diabetes mellitus”[Title/Abstract] OR T1D[Title/Abstract] OR T1DM[Title/Abstract]) AND (adolescen*[Title/Abstract] OR child*[Title/Abstract] OR teen*[Title/Abstract] OR youth*[Title/Abstract] OR pediatric*[Title/Abstract])) AND (psychosocial*[Title/Abstract] OR psycholog*[Title/Abstract] OR communicat*[Title/Abstract] OR “self confidence”[Title/Abstract] OR “self efficacy”[Title/Abstract] OR “self esteem”[Title/Abstract] OR distress*[Title/Abstract] OR “fear of hypoglyc*"[Title/Abstract] OR acceptance[Title/Abstract] OR autonom*[Title/Abstract] OR hope*[Title/Abstract] OR optimis*[Title/Abstract] OR motivat*[Title/Abstract] OR burnout[Title/Abstract] OR normaliz*[Title/Abstract] OR “illness identity”[Title/Abstract] OR “illness perception*”[Title/Abstract] OR “patient provide*”[Title/Abstract] OR “patient center*”[Title/Abstract] OR “provider patient”[Title/Abstract] OR stigma*[Title/Abstract] OR “social isolation”[Title/Abstract] OR “social support”[Title/Abstract] OR “family conflict”[Title/Abstract])) AND (survey* OR questionnaire* OR scale* OR quantitative OR measure* OR intervention* OR experiment* OR “empirical study” OR “prospective study” OR “follow up study” OR “longitudinal study” OR “retrospective study” OR “clinical trial”)) NOT (gestation*[Title/Abstract] OR pregnan*[Title/Abstract])

**All EBSCO Databases (Academic Search Premier, CINAHL, PsycInfo, Global Health)**

AB ( (“type 1 diabet*” OR “insulin dependent diabetes” OR IDDM OR “juvenile diabetes” OR “diabetes mellitus” OR T1D*) ) AND AB ( (adolescen* OR child* OR teen* OR youth* OR pediatric*) ) AND AB ( (psychosocial* OR psycholog* OR communicat* OR “self confidence” OR “self efficacy” OR “self esteem” OR distress* OR “fear of hypoglyc*” OR acceptance OR autonom* OR hope* OR optimis* OR motivat* OR burnout OR normaliz* OR “illness identity” OR “illness perception*” OR “patient provide*” OR “patient center*” OR “provider patient” OR stigma* OR “social isolation” OR “social support” OR “family conflict”) ) AND ( (survey* OR questionnaire* OR scale* OR quantitative OR measure* OR intervention* OR experiment* OR “empirical study” OR “prospective study” OR “follow up study” OR “longitudinal study” OR “retrospective study” OR “clinical trial”) ) NOT AB ( (gestation* OR pregnan*) )

**LWW Nursing —**

((("type 1 diabet*" or "insulin dependent diabetes" or IDDM or "juvenile diabetes" or "diabetes mellitus" or T1D*) not gestation* not pregnan*) and (adolescen* or child* or teen* or pediatric*) and (psychosocial* or psycholog* or communic* or "self confidence" or "self efficacy" or “self esteem” or distress or “fear of hypoglyc* or acceptance or autonom* or hope* or optimis* or motivat* or burnout or normaliz* or “illness identity” or “illness perception*” or "patient provide*" or "patient center*" or "provider patient" or stigma or "social isolation" or "social support" or “family conflict”)).ab. and (survey or questionnaire or scale or quantitative or measure* or intervention* or experiment* or "empirical study" or "prospective study" or "follow up study" or "longitudinal study" or retrospective or "clinical trial").af

**Web of Science —**

((AB=("type 1 diabet*"  OR  "insulin dependent diabetes"  OR  IDDM  OR  "juvenile diabetes"  OR  "diabetes mellitus"  OR T1D*)  AND  AB=(adolescen*  OR  child*  OR  teen*  OR  youth*  OR  pediatric*)  AND  AB=(psychosocial*  OR psychological*  OR communic*  OR  "self confidence"  OR "self efficacy"  OR  acceptance  OR  "illness perception*"  OR hope*  OR  optimis*  OR  motivat*  OR  autonomies*  OR distress*  OR  normaliz*  OR  "illness identity"  OR  "patient provide*"  OR  "patient center*"  OR "provider patient"  OR  stigma  OR  "self consciousness"  OR "self esteem"  OR "social isolation"  OR  "social support"  OR  burnout  OR  "family conflict")  AND KP=(survey  OR  questionnaire  OR  scale  OR  quantitative  OR  measure*  OR  intervention*  OR experiment*  OR "empirical study"  OR "prospective study"  OR "follow up study"  OR "longitudinal study"  OR "retrospective study"  OR "clinical trial")  NOT AB=(gestation*  OR pregnan*))) *AND* **LANGUAGE:** (English)

**Embase —**

('type 1 diabet*':ti,ab,kw OR 'insulin dependent diabetes':ti,ab,kw OR iddm:ti,ab,kw OR 'juvenile diabetes':ti,ab,kw OR 'diabetes mellitus':ti,ab,kw OR t1d*:ti,ab,kw) AND (adolescen*:ti,ab,kw OR child*:ti,ab,kw OR teen*:ti,ab,kw OR youth*:ti,ab,kw OR pediatric*:ti,ab,kw) AND (psychosocial*:ti,ab,kw OR psycholog*:ti,ab,kw OR communicat*:ti,ab,kw OR 'self confidence':ti,ab,kw OR 'self efficacy':ti,ab,kw OR 'self esteem':ti,ab,kw OR distress*:ti,ab,kw OR 'fear of hypoglyc*':ti,ab,kw OR acceptance:ti,ab,kw OR autonom*:ti,ab,kw OR hope*:ti,ab,kw OR optimis*:ti,ab,kw OR motivat*:ti,ab,kw OR burnout:ti,ab,kw OR normaliz*:ti,ab,kw OR 'illness identity':ti,ab,kw OR 'illness perception*':ti,ab,kw OR 'patient provide*':ti,ab,kw OR 'patient center*':ti,ab,kw OR 'provider patient':ti,ab,kw OR stigma*:ti,ab,kw OR 'social isolation':ti,ab,kw OR 'social support':ti,ab,kw OR 'family conflict':ti,ab,kw) AND (survey*:ti,ab,kw OR questionnaire*:ti,ab,kw OR scale*:ti,ab,kw OR quantitative:ti,ab,kw OR measure*:ti,ab,kw OR intervention*:ti,ab,kw OR experiment*:ti,ab,kw OR 'empirical study':ti,ab,kw OR 'prospective study':ti,ab,kw OR 'follow up study':ti,ab,kw OR 'longitudinal study':ti,ab,kw OR 'retrospective study':ti,ab,kw OR 'clinical trial':ti,ab,kw) NOT (gestation*:ti,ab,kw OR pregnan*:ti,ab,kw) AND [2010-2020]/py
