# Supplementary material for: MeSH and text-word search strategies: precision, recall, and their implications for library instruction
Source: J Med Libr Assoc. 2022 Jan 1;110(1):23–33. doi: 10.5195/jmla.2022.1283 (PMC8830400; doi:10.5195/jmla.2022.1283)
Supplement: Supplementary file 3 — Appendix C. Comparison of MeSH terms used [file jmla-110-1-23-s03.docx]

**Appendix C: Comparison of MeSH Terms Used**

| **PubMed**  **Text-Words** | **PubMed**  **MeSH Term** | **CINAHL**  **MeSH Term** | **PsycInfo**  **MeSH Term** | **Embase**  **Emtree Term** |
| --- | --- | --- | --- | --- |
| “type 1 diabet*” OR “insulin dependent diabetes” OR IDDM OR “juvenile diabetes” OR “diabetes mellitus” OR T1D OR T1DM | Diabetes Mellitus, Type 1 | Diabetes Mellitus, Type 1 | Diabetes Mellitus, Type 1 | Insulin Dependent Diabetes |
| adolescen* OR child* OR teen* OR youth* OR pediatric* | Child OR Adolescent | Child OR Adolescence | Child OR Adolescent | Child OR Adolescent |
| Acceptance | Patient Acceptance of Health Care | n/a | Patient Acceptance of Health Care | Patient Acceptance of Health Care |
| self efficacy OR self confidence OR self esteem OR autonomy* | Self Efficacy OR Self Concept | Self Efficacy OR Self Concept | Self Efficacy OR Self Concept | Self Concept |
| psychosocial* OR psycholog* | Psychology OR Psychology, Adolescent | Psychology OR Psychology, Adolescent | Psychology, Adolescent | Psychology OR Child Psychology |
| communicat* | Teach-Back Communication OR Health Communication | n/a | Teach-Back Communication OR Health Communication | Interpersonal communication OR Medical Information |
| distress | Psychological Distress | Psychological Distress | Psychological Distress | Distress Syndrome |
| n/a | Uncertainty^[[1]](#footnote-1)^ | Uncertainty | Uncertainty | Uncertainty |
| hope | Hope | hope | Hope | Hope |
| optimis* | Optimism | Optimism | Optimism | Optimism |
| stigma | Social Stigma | Social Stigma | Social Stigma | Social Stigma |
| social isolation | Social Isolation | Social isolation | Social Isolation | Social Isolation |
| social support | Psychosocial Support Systems | Support, Psychosocial | Social Support OR Psychosocial Support Systems | Social Support OR Psychosocial care |
| burnout | Burnout, Psychological | n/a | Burnout, Psychological | Burnout |
| n/a | Self Care^[[2]](#footnote-2)^ | Self Care | Self Care | Self Care |
| fear of hypoglyc* | Fear | Fear | Fear | Fear |
| “patient center*” | Patient-Centered Care | Patient Centered Care | Patient-Centered Care | Patient care |
| ‘family conflict” | Family Conflict | Family conflict | Family Conflict | Family Conflict |
| “illness perception” OR “illness identity” | Perception | Perception | Perception | Perception |
| “patient provide*” OR “provider patient” | Professional-Patient Relations | Professional-Patient Relations | Professional-Patient Relations | Professional-patient relationship |
| motivate* | n/a^[[3]](#footnote-3)^ | n/a | n/a | n/a |
| Normalize* | n/a | n/a | n/a | n/a |

1. Used as a subcategory of Psychological Phenomena [↑](#footnote-ref-1)
2. Should have limited to Self Care/Psychology [↑](#footnote-ref-2)
3. Could have used Motivation/Psychology or Power, Psychological Empowerment [↑](#footnote-ref-3)
